# Posterior Reversible Encephalopathy in Undiagnosed Systemic Lupus Erythematosus: A Rare Case Report

**DOI:** 10.7759/cureus.16945

**Published:** 2021-08-06

**Authors:** Wardah Khalid Rafat, Shah T Sarmast, Saher T Shiza, Kehinde Olaleye, Sylvette Rogers

**Affiliations:** 1 Internal Medicine, Ziauddin University, Karachi, PAK; 2 Neurology, California Institute of Behavioral Neuroscience and Psychology, California, USA; 3 Internal Medicine, Deccan College of Medical Sciences, Hhyderabad, IND; 4 Neurological Sciences, Tampa General Hospital, Tampa, USA; 5 Family Medicine, Caribbean Medical University, Des Plaines, USA

**Keywords:** seizure, posterior reversible encephalopathy syndrome, systemic lupus erythematosus, pres, sle

## Abstract

Systemic lupus erythematosus (SLE) is a multisystem autoimmune disorder known to affect the nervous system by direct neuronal damage, vasculitis, or pathologic mechanisms indirectly induced by immune mechanisms related to the production and deposition of immune complexes. SLE has a wide range of clinical manifestations due to the involvement of almost every organ system of the body. SLE presents with serositis, mucositis, arthralgia, glomerulopathy, hematological, cutaneous, and hematological manifestations. Among the neurological manifestations of SLE, posterior reversible encephalopathy is rarely described in the literature. We report a case of posterior reversible encephalopathy in a female patient who presented with seizures, altered mentation, headache, and blurry vision in the setting of undiagnosed SLE.

## Introduction

Systemic lupus erythematosus (SLE) is a connective tissue autoimmune disease characterized by a broad spectrum of clinical manifestations. It commonly presents with arthritis, glomerulonephritis, serositis, cutaneous, hematological, and nervous system manifestations [1]. Neurological manifestations include headache, seizures, movement disorder, transient ischemic attack, acute confusional state, dementia, and cerebellar syndromes [2]. The prevalence of neurological manifestations in SLE is highly variable, ranging from 18%-67% [2]. Posterior reversible encephalopathy syndrome (PRES) as an initial manifestation of SLE is not widely reported in the literature. PRES is a clinical syndrome presenting various neurological manifestations, including headaches, seizures, altered mentation, and visual changes [3]. A case-control study reported a prevalence of PRES in SLE as much as 0.43% [3]. We report a case of SLE presenting with seizure as an initial manifestation of PRES in a female patient.

## Case presentation

A 31-year-old female with a past medical history of hypertension was brought to the emergency department with sudden onset convulsive episode. The seizure was generalized and associated with urinary incontinence, upward rolling of eyes balls, and tongue bite, followed by poor speech and altered mentation. She had a history of generalized weakness, fatigue, episodic headache, and blurry vision for the last two weeks. She did not drink alcohol and use any illicit drugs. She had no history of cough, nausea, vomiting, weight loss, seizures, trauma, and travel.

**Figure 1:**
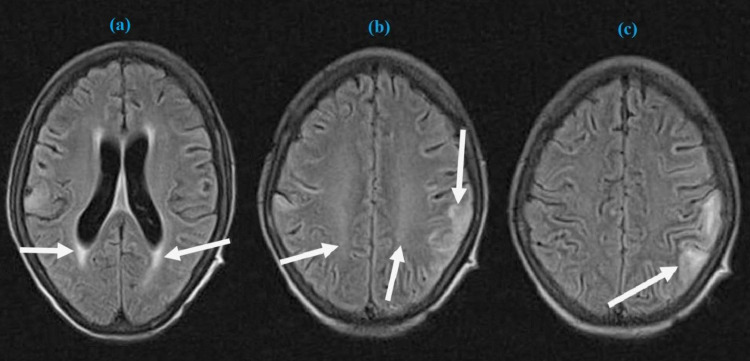
Axial images of brain MRI showing patchy areas of hyperintense signals (white arrows) in cortical and subcortical areas of temporal, parietal (a, b), and occipital lobes (c). MRI: magnetic resonance imaging.

On examination, she looked confused and anxious with incoherent speech. She had a temperature of 99^o^F, blood pressure of 170/110 mmHg, respiratory rate of 23/minute, and oxygen saturation of 95%. Her Glasgow Coma Scale (GCS) was 11/15. She had a tongue bite mark and bilaterally normal pupils reactive to light with normal fundoscopy. Cranial nerves were intact on examination. The erythematous and scaly rash was evident on the forehead. The rest of the neurological examination was unremarkable. Clinical examination of the other systems did not reveal any abnormality. Her initial blood workup revealed anemia and thrombocytopenia. Elevation of C-reactive protein and erythrocyte sedimentation rate was also observed. Chest X-ray was unremarkable. Brain magnetic resonance imaging (MRI) was performed, which revealed patchy areas of hyperintense signals in cortical and subcortical areas of temporal, parietal, and occipital lobes indicative of PRES (Figures 1a-1c). She underwent cerebrospinal fluid (CSF) analysis, and the result of CSF analysis is shown in Table 1.

**Table 1:** Results of cerebrospinal fluid (CSF) analysis.

Spinal fluid	Lab values	Reference range
Color	Colorless	Colorless
Protein	132	15-45 mg/dL
Opening pressure	13	05-20 cmH_2_0
Red blood cell count	69	0 mm^3^
White blood cell count	04	0-05 mm^3^
Glucose	135	40-70 mg/dL
Gram stain	Negative	Negative
Bacterial antigen	Negative	Negative

Blood and CSF cultures did not show any organism. Initially, she was managed with intravenous phenytoin with a loading dose of 10mg//kg, and blood pressure was controlled with intravenous labetalol. Her condition improved gradually, and she was symptom-free on day five with normal speech. On further inquiry, she also informed that she had repeated painful oral ulceration for the last two months, and intermittent polyarthralgia lasting four to five days, for the last one year and not affecting her daily activities. She had no dysuria, hematuria, leg edema, photosensitivity, and abnormal hair loss. On subsequent examination, she had a scaly and erythematous rash on the anterior aspect of both legs. In addition, oral ulcers were present on the buccal mucosa and hard palate. The diagnosis favored SLE considering the history and clinical findings of musculoskeletal and other system examinations. The autoimmune screening was performed, and the results are shown in Table 2.

**Table 2:** Results of autoimmune screening. Anti-dsDNA Ab: anti-double-stranded deoxyribonucleic acid antibody, ant-CCP: anti-cyclic citrullinated peptide, anti-β2GP1: anti-beta-2 glycoprotein 1, ANCA: antineutrophil cytoplasmic antibodies.

Parameter	Lab value	Reference range
Anti-dsDNA Ab (IU/mL)	61	< 35
Complement C3 protein (mg/dL)	81	90-100
Complement C4 protein (mg/dL)	09	10-40
Anti-CCP (IU/mL)	39	< 20
Anticardiolipin	Negative	Negative
Lupus anticoagulant	Negative	Negative
Anti-β2GP1 Ab	Negative	Negative
ANCA	Negative	Negative

She met the diagnostic criteria of SLE and was diagnosed with SLE based on history, clinical findings, and laboratory investigations. She was put on hydroxychloroquine and low-dose glucocorticoids and was discharged on day seven on follow-up with her primary care physician.

## Discussion

PRES in SLE commonly affects the female, with a median age of onset ranging from 9-82 years. PRES is generally triggered by autoimmune disease, hypertension, eclampsia, drug abuse, and kidney disease [4]. Fugate et al. highlighted the prevalence of signs and symptoms in 113 patients with PRES and reported that seizure was the most common symptom, followed by encephalopathy, headaches, and visual disturbances [5]. However, data on seizures as an initial manifestation of SLE is lacking. Iftikhar et al. underlined a case presented with seizures as an initial presentation of SLE [6]. Benavente et al. reported a case of SLE in a patient who presented with seizures and encephalitis [7]. Our patient presented with headaches, seizure episodes, and confusion as a PRES syndrome leading to SLE diagnosis.

Seizures are the critical manifestations of neuropsychiatric SLE, and a total of 10%-20% of SLE patients experience seizures at some stage of SLE [8]. The incidence of epilepsy in SLE is eight times higher in SLE patients than in general patients, and mild seizures have been reported in around 5%-10% of the patients, which can develop even before the clinical onset of the disease [9]. Another study reported three times more prevalence of seizures in SLE patients as compared to the general population. Andrade et al. reported that 40 patients developed seizures among 600 SLE patients with a prevalence of 6.7% [10].

Generally, the patients develop seizures in the context of active systemic disease; however, it can be an isolated finding. Pathogenesis of seizures and PRES is often multifactorial. It may involve inflammatory cytokines, autoantibodies production, premature atherosclerosis, microangiopathy and nonspecific immune complexes, and vasogenic edema in the context of hypertension that accelerates neuronal damage [1,11]. A study highlighted that advanced PRES in SLE results from endothelial cells injury, vasoconstriction, and immune-mediated damage to neurons [4]. Another mechanism may be disruption of the blood-brain barrier resulting from abnormal endothelial and white blood cell interactions facilitating the lymphocyte entry into the nervous system [1].

Our patient was diagnosed with SLE based on the American College of Rheumatology (ACR) criteria [12]. The pattern of central nervous system involvement in lupus could be focal, diffuse, or a combination of both, serving as a diagnostic challenge for the physicians [2]. Therefore, an extensive differential diagnosis should be considered in patients presenting with seizures. ACR criteria outlined the guidelines for a comprehensive approach, workup, and appropriate management of each clinical condition. Primary seizure disorder, cerebral ischemia, encephalitis, meningitis, and lupus cerebritis are among SLE differential diagnoses, and ACR criteria do not include PRES syndrome, a diagnosis of exclusion with relevant supportive findings [12].

PRES syndrome and lupus cerebritis have the same clinical presentation; however, both are different clinical entities due to CSF antibodies and nonspecific changes on MRI of the brain in lupus cerebritis. In addition, vasogenic edema is more specific to PRES syndrome and usually resolves within few days of appropriate management [13]. Therefore, for a definitive diagnosis of SLE, the patients must undergo complete clinical and laboratory workup to exclude any other autoimmune condition. Management of SLE includes symptomatic treatment, immunosuppressive, and biological agents, and therapy is individualized based on functional status, clinical presentation, and disease severity [11].

## Conclusions

SLE is a multisystem disease affecting nearly every organ system of the body, making its diagnosis difficult, especially when the patient presents with rare manifestations. Although PRES is not common in SLE, it should be considered among differential diagnoses in patients presenting with seizures, altered mentation, and blurry vision consistent with brain imaging. This case highlights the imperative assessment, early diagnosis, and treatment of PRES in SLE, and delayed management can lead to severe morbidity and mortality. Due to varying clinical presentation, and involvement of several organ systems in SLE, there is a dire need to explore the neurological manifestations for the possibility of SLE in the absence of other definitive diagnoses.
